# High-Precision, Wide-Ratio, Self-Balancing Current Comparator

**DOI:** 10.3390/s25175289

**Published:** 2025-08-26

**Authors:** Xue Wang, He Li, Hao Liu, Xunan Ding, Jingang Wang, Pengcheng Zhao, Hongbiao Jiang

**Affiliations:** 1 China Electric Power Research Institute, Wuhan 430074, China; wangxue@epri.sgcc.com.cn (X.W.); hhlh0@163.com (H.L.); 2 State Grid Zhejiang Marketing Service Center, Hangzhou 310007, China; liberty1120@126.com; 3 State Key Laboratory of Power Transmission Equipment Technology, School of Electrical Engineering, Chongqing University, Chongqing 400044, China; jingang@cqu.edu.cn (J.W.); zhaopengcheng@cqu.edu.cn (P.Z.); 4 State Grid Jinhua Power Supply Company, Jinhua 321017, China; jhb_2001_938@163.com

**Keywords:** zero-flux, wide-ratio, self-balancing, current comparator, automatic measurement

## Abstract

The current proportional standard device is the most important equipment in the traceability system of current proportional values. Due to the influence of magnetic and capacitive errors, existing current proportional standard devices have the characteristics of high precision and wide variation ratio. This article focuses on the current comparator based on the zero magnetic flux principle, and combines electronic compensation technology with magnetic and capacitive error analysis to develop a new type of wide-variable-ratio self-balancing current comparator. It can achieve many ratio transformations while maintaining high accuracy levels. It can be used to calibrate high-precision standard currents of 0.001 level and below, greatly improving work efficiency. The principle and structure of the wide-variable-ratio self-balancing current comparator are described in the article, and its error performance is theoretically analyzed. Error calibration experiments are also conducted. The test results show that the accuracy of the current comparator with a wide range ratio meets the requirement of 0.0002 level, which can be used to solve the problem of high accuracy and low efficiency in wide range current ratio calibration tests.

## 1. Introduction

A current transformer is a current proportional instrument whose current ratio is inversely proportional to its number of turns. Due to the secondary load of the current transformer, the iron core requires an excitation current. The negative value of the ratio of the primary excitation current to the primary current phasor is the complex error of the current transformer. The current ratio standard device, as a device for verifying the error of current transformers, has a history of over 100 years of application, mainly including two-stage current transformers, zero-flux current transformers, and self-balancing current transformers [1,2,3].

The two-stage current transformer uses an auxiliary iron core to bear most of the load, making the main iron core load small and the magnetic flux low. In addition, capacitor compensation significantly reduces the error. Shanxi Transformer Factory has successfully developed a high-precision two-stage current transformer, which significantly reduces the error of the current transformer and can achieve an accuracy level of 0.001. Later, research was conducted on compensation based on this, and the magnetic potential compensation method and electric potential compensation method were proposed, which gradually matured in theory. In 1961, Canadian Kusters proposed a compensating current comparator, which uses a compensating winding wound only on the main iron core and not on the shielding iron core, connected in parallel with the secondary winding, so that the induced potential generated by the shielding iron core in the secondary winding automatically bears the additional load, and the problem of increasing load was satisfactorily solved [4,5,6,7]. Prior to this, standard current transformers were generally used in the fields of quantity traceability and bridge technology. The decisive step in the development of current comparators was the introduction of compensation windings. Based on this principle, the 60,000 A power/frequency current ratio standard developed by the National High Voltage Metrology Station and Tianjin Transformer Factory adopts traditional electromagnetic compensation, with an error of no more than 0.2 × 10^−7^. It is stored in the National High Voltage Metrology Station and has become the standard device with the highest accuracy level for power frequency high current ratio. With the development of electronic technology, active self-balancing has emerged, which is more convenient to use and has greatly expanded its application fields. O. Petersons found that at 400 Hz, the ratio and angle difference of 100 A/1 A are both less than 1 ppm [8]. T. Michael Souder from the National Bureau of Weights and Measures in the United States introduced a broadband current transformer developed by him, with a primary current of 5 A~100 A and a secondary current of 5 A. The frequency ranges from 50 Hz to 10 kHz, with an error of less than 1 ppm below 1 kHz [9,10].

A two-stage current transformer is composed of two current transformers, and the error is determined by the excitation turns of the second-stage transformer core, which is the product of the errors of the first- and second-stage transformers. However, the accuracy level is relatively low, generally only up to 0.001 level, which cannot meet the requirements of high-precision current ratio conversion. The wiring is complex, and the compensation winding is connected in parallel with the secondary winding, and the number of turns must be equal. Changing the ratio wiring is cumbersome. The compensating current comparator uses a compensating winding that is only wound on the main iron core and not on the shielding iron core. It is connected in parallel with the secondary winding and uses the induced potential generated by the secondary winding on the shielding iron core to supply power to the additional impedance [11,12]. Its accuracy level is high and can reach the ppm level, but it is complex to use. Since it cannot generate the secondary current to be compared, it needs to be injected from the outside. When using it, an auxiliary transformer needs to be configured to achieve a zero-magnetic-flux state. The zeroing process is complicated and the measurement range is narrow. In order to improve the accuracy of the current comparator, the transformation ratio design of a single current comparator will not exceed 15. The higher the accuracy level, the fewer the transformation ratios. With the development demand of digital transformation of measurement standards and measuring instruments and equipment, high-precision measuring equipment is gradually shifting towards digitization and intelligence. To achieve fully automatic measurement of high-precision current ratios, it is still necessary to use a self-balancing current comparator based on auxiliary circuits. However, the current comparator compensated by existing auxiliary circuits cannot meet the requirements of both width-to-width ratio and high precision [13,14,15,16,17,18,19].

In response to the above problems and shortcomings, a high-precision and wide-variable-ratio self-balancing current comparator is developed, with a current range from 5 A to 5000 A and secondary outputs of 5 A and 1 A. The accuracy level can reach 0.0002 levels or above. This article first briefly describes the principle of traditional current comparator, and based on this, theoretically analyzes its error sources and conducts corresponding calibration tests. Its wide application can greatly improve the efficiency of traceability and transmission of existing high-precision current ratio standard devices, providing favorable support for the intelligent digital transformation of high current precision measurement [20].

## 2. Principles

The current comparator operates in the zero-flux state and does not require an excitation current, which might lead to the assumption that it is free from errors. However, research indicates that the current comparator still exhibits errors, specifically the magnetic error ε_L_ and the capacitive error ε_C_, though these differ from the errors generated by an excitation current in conventional current transformers.

The current comparator utilizes the induced potential generated by the secondary winding on the shielded core to supply an additional impedance. Unlike traditional designs, it does not require a separate excitation winding or an external power source. Instead, it directly employs a compensating winding N_B_, wound solely on the main core and connected in parallel with the secondary winding N_2_. Crucially, the turn ratio must satisfy N_B_ = N_2_.

**Figure 1:**
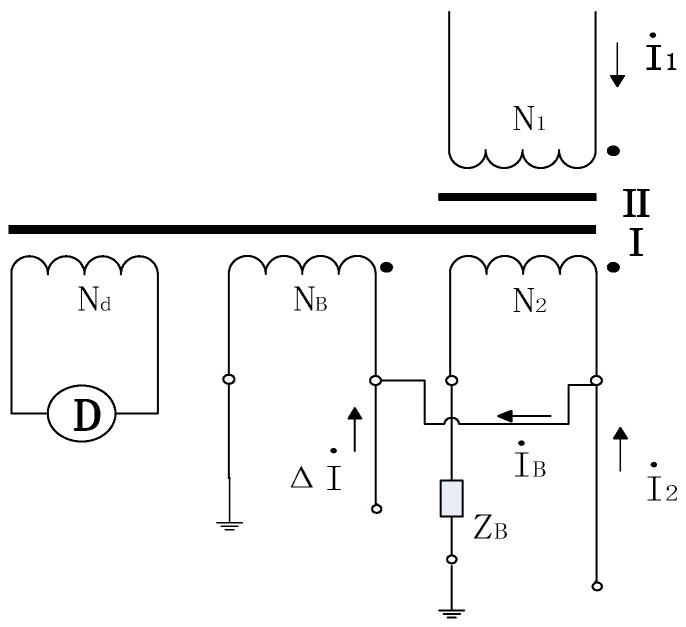
Principle diagram of compensated current comparator. N_1_, N_2_: Number of turns in the primary and secondary windings, respectively. N_B_: Number of turns in the compensation winding. *I*_1_, *I*_2_: Primary and secondary currents, respectively. N_d_: Number of turns in the detection winding. ΔI: Error current. *I*_B_: compensation current. Z_B_: Load impedance.

The schematic diagram (Figure 1) illustrates the principle: Core I serves as the main core, while Core II acts as the shielded core. The induced voltage generated by N_2_ on the shielded core is given by the following formula:

(1) $$E_{b}=I_{2}Z_{2}-I_{B}\left(Z_{2}+Z_{B}\right)\approx I_{2}Z_{2}$$

If the proportional windings N_1_, N_2_ and the shielding core are considered together as an auxiliary transformer, then *I*_B_/*I*_2_ represents the complex error of the auxiliary transformer, where *I*_B_ is much smaller than *I*_2_. When used as a current comparator for downward value transfer, an auxiliary transformer is typically required to first adjust it to a zero-flux state before conducting the value transfer test. Drawing on the principle of the compensated current comparator, the excitation current is amplified via an electronic circuit. Through the design of compensation circuit and the adjustment of closed-loop feedback control parameters, high accuracy and sufficient stability can be achieved.

**Figure 2:**
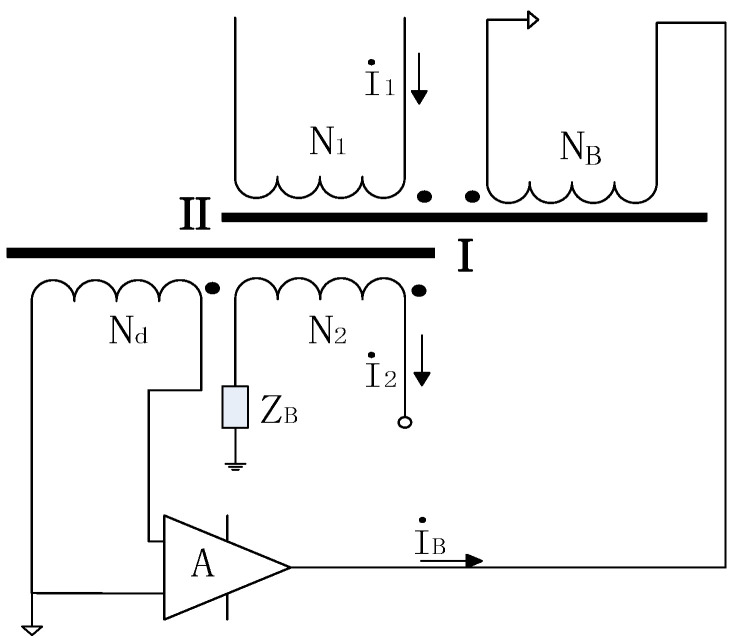
Principle diagram of a high-precision, wide-ratio, self-balancing device.

The schematic diagram of the current comparator is shown in Figure 2. To minimize the influence of leakage flux from the ratio windings N_1_, N_2_ or external stray magnetic fields on the main core, a shielding core is added outside the detection winding. The output of the detection winding is connected to an amplifier circuit A, whose output drives the compensation winding N_B_. The detection winding N_d_ measures the magnetic flux in the main core, and the compensation current *I*_B_ injected into the compensation winding N_B_ is adjusted via the amplifier to maintain the main core in a near-zero-flux state. Since the compensation current does not act on the main core, the detection voltage is proportional to the ampere-turn imbalance of the ratio windings.

The detection voltage, after amplification by the electronic circuit, energizes the shielding core to induce the secondary current. Due to the extremely high gain of the amplifier, the detection voltage approaches zero, meaning the ampere-turn difference between the ratio windings also nears zero. Consequently, the primary and secondary currents flowing through the ratio windings maintain a strict inverse relationship with their turn ratio.

In essence, this system employs an amplifier to compensate for the error current generated in the ratio windings, achieving automatic magnetomotive force balance.

(2) $$I_{1}N_{1}+I_{2}N_{2}\approx 0$$

## 3. Error Analysis

The current comparator is often assumed to be error-free because it operates in a zero-flux state, requiring no excitation current. However, research shows that current comparators still exhibit errors. In addition to magnetic and capacitive errors, the self-balancing current comparators with a wide-ratio range also introduces gain errors. The mechanisms of magnetic and capacitive errors are the same as those in traditional compensated current comparators and will not be reiterated here. Instead, this paper focuses on the design improvements for the new self-balancing current ratio standard to address magnetic and capacitive errors, with particular emphasis on the derivation and suppression measures for gain errors.

### 3.1. Magnetic Error

The magnetic error of the current comparator is mainly affected by the following factors. Firstly, the low-frequency current comparator detects the nonlinearity of the iron core material, which leads to uneven magnetic permeability at various locations of the iron core under the action of an uneven magnetic field. At the same time, uneven winding and heat treatment of the iron core can also lead to uneven magnetic permeability at various locations of the annular iron core. Secondly, the primary winding and secondary winding are not fully coupled, and the uneven winding of the detection winding leads to magnetic leakage. When a large current flows through the primary winding of the three comparators during operation, the return conductor will generate an interference magnetic field, and the surrounding electrical equipment will also generate an interference magnetic field. The interference magnetic field enters the detection iron core of the current comparator, causing magnetic errors [21].

By designing magnetic shielding, most of the magnetic flux leakage can be reflected in the magnetic shielding, greatly reducing the magnetic flux leakage of the detection iron core. Therefore, magnetic shielding is also the most effective means to reduce magnetic errors. The reason why magnetic flux leakage reduces the accuracy of the current comparator is that it causes the induced voltage generated in the detection coil to no longer be evenly distributed along the path of the detection iron core [12,13,14,15,16,17,18,19,20,21,22,23]. Through research, it is known that increasing the thickness of magnetic shielding can help improve magnetic flux non-uniformity. When the thickness of magnetic shielding is the same, the higher the relative magnetic permeability, the better the shielding effect. As the effect of increasing the thickness of the magnetic shielding to a certain extent on improving the accuracy of the current comparator gradually weakens, it is necessary to try other methods to further improve the accuracy of the current comparator. Adopting a double-layer magnetic shielding structure and observing whether it can better reduce magnetic flux non-uniformity compared to single-layer shielding of the same thickness [24].

**Figure 3:**
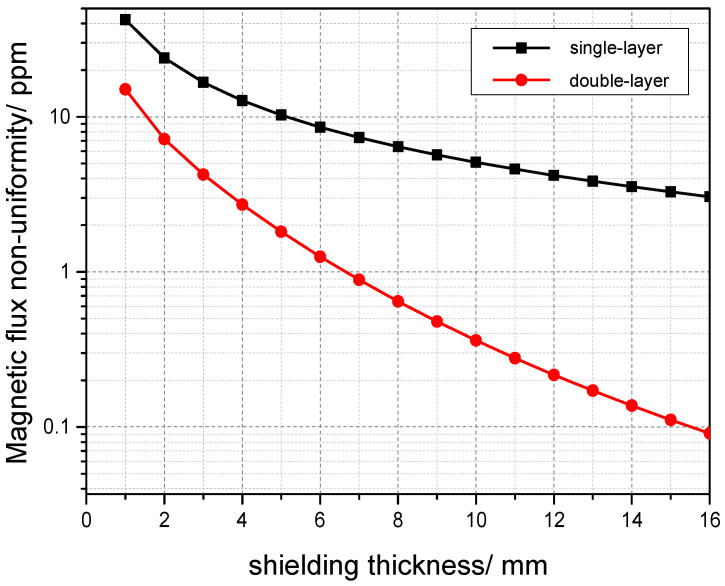
Comparison of shielding effects between single-layer shielding and double-layer shielding.

Selecting 10 mm thick permalloy as a single-layer magnetic shield, a double-layer is equivalent to stacking two single layers. Using analytical methods for calculation, the shielding effects of single-layer shielding and double-layer shielding are compared. Under the conditions of constant relative magnetic permeability and thickness, using 10 mm double-layer magnetic shielding can significantly reduce magnetic flux non-uniformity and improve the shielding effect [25].The simulation results are shown in Figure 3.

We verify the measurement results of the analytical method through magnetic shielding effectiveness testing. Generally, the shielding efficiency is used to express the degree of weakening of the magnetic shielding to the inhomogeneous magnetic field, and it can also be characterized by the magnitude of the voltage, the greater the shielding effect, the better the shielding effect, and the expression is as follows:

(3) $$S=\frac{H_{0}}{H}=\frac{U_{0}}{U}$$

where *S* is the shielding coefficient, *H*_0_ is the field strength of a point on the axis of the iron core when there is no magnetic shielding, and *H* is the field strength of the point when there is a magnetic shield, and the field strength can be characterized by the magnitude of the voltage. The number 0 represents bare iron core, 1 represents adding one layer of magnetic shielding, 2 represents adding another layer of copper shielding, and 3 represents adding another layer of magnetic shielding. The measurement results are shown in Table 1 [26,27].

As the number of shielding layers increases, the induced voltage decreases, and the error caused by leakage flux interference on the main iron core also decreases. After adding a comprehensive magnetic shielding, the impact of magnetic leakage was reduced by 57 dB, about 1500 times [28].

The control of magnetic errors primarily relies on two measures: effective magnetic shielding and high spatial coincidence of proportional windings. Based on existing research achievements in magnetic shielding, a well-designed magnetic shield was implemented for the detection winding, and magnetic field interference tests were conducted. By applying external interference magnetic fields, either from a dipole or external current with a uniform intensity of 10 AT, the output voltage of the detection winding remained below 5 μV. This indicates that the error induced by the interference magnetic field is less than 1 μAT, corresponding to an error of 0.02 × 10^−6^ under rated current conditions. Therefore, it is reasonable to conclude that the impact of magnetic errors is below 0.02 × 10^−6^.

### 3.2. Capacitive Error

In a current comparator, distributed capacitance exists between turns, layers, windings, and each winding to ground. When the operating winding carries current, the resulting impedance voltage drop is applied across this distributed capacitance, generating capacitive currents that flow through the windings and introduce errors. Such errors caused by capacitive currents are referred to as capacitive errors.

In the development of a high-precision, wide-ratio, self-balancing current comparator, the following design considerations can be adopted to minimize capacitive errors:

- Minimize Winding Turns

The ampere-turns of the comparator should be kept appropriately low. However, excessively low ampere-turns may increase magnetic errors, particularly reducing immunity to external magnetic interference and zero-adjustment stability. Therefore, the selection of ampere-turns must balance capacitive and magnetic errors to achieve an overall minimum.

- Optimal Winding Arrangement

Generally, the method of uniformly winding one or several layers of each winding along the circumference of the iron core is adopted. In order to reduce the potential difference between the windings, the windings are wound in sequence. If wound arbitrarily, the potential difference between the windings increases after series connection, and the capacitance error increases. For a single winding without a middle tap, it can be wound in sections. The principle is to maximize the utilization rate of the iron core when the comparator is working.

- Parallel or Segmented Idle Windings

In current comparators, idle windings are often eliminated using series-parallel structures, ensuring all windings participate in operation at each current ratio. If the current ratios are integer multiples and the winding arrangement is well-organized, parallel connection significantly reduces capacitive errors. However, for multiple current ratios and high ampere-turns, parallel connection may increase capacitive errors and complicate wiring. Thus, in multi-ratio comparators, segmented windings (typically ≤ 100 or 200 turns) are preferred, where idle windings are disconnected from primary or secondary windings to minimize capacitive errors.

- Reducing Winding Layers

For current ratios *n* > 1 and moderate turn counts, single-layer uniformly distributed windings can be used without parallel or series connections. To avoid excessive size, standalone windings should be limited. Since such windings are uniformly distributed in a single layer, both capacitive and magnetic errors remain small.

- Electrostatic Shielding

Grounded electrostatic shielding can be employed to isolate windings layer by layer, preventing mutual interference and stabilizing capacitive errors. This facilitates independent control over individual windings.

### 3.3. Gain Error

**Figure 4:**
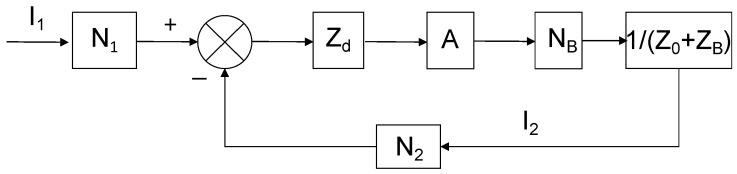
Steady-state working principle block diagram of a wide-ratio self-balancing current comparator. N_1_, N_2_: Number of turns in the primary and secondary windings, respectively. N_B_: Number of turns in the compensation winding. *I*_1_, *I*_2_: Primary and secondary currents, respectively. Z_d_: Sensitivity of the detection winding, unit: Ω. A: Voltage gain of the auxiliary amplifier circuit, unit: V/V. Z_0_: Internal resistance and leakage reactance of the secondary winding. Z_B_: Load impedance.

In traditional theory, the gain error is not considered for compensated current comparators. However, it is inherently reflected in the sensitivity index. A lower sensitivity indicates a weaker response capability of the current comparator to ampere-turn differences. In this case, even if the null detector displays zero, it does not necessarily mean that the two compared ampere-turns are equal. For the wide-ratio self-balancing compensated current comparator developed in this paper, the gain error is of great significance. By modeling the steady-state working principle with reference to control theory, Figure 4 illustrates the operational block diagram. The bold and italic fonts in Figure 4 represent vectors or complex quantities. Through this working principle analysis, the mechanism of gain error generation can be clearly understood.

Assuming magnetic and capacitive errors are negligible, the working principle is described in detail as follows:

The combined ampere-turns (*I*_1_N_1_ − *I*_2_N_2_) of the primary current *I*_1_ and secondary current *I*_2_ excite the main core, inducing a voltage in the detection winding as follows:

(4) $$U_{d}=\left(I_{1}N_{1}-I_{2}N_{2}\right)Z_{d}$$

and further simplification can be achieved.

(5) $$\varepsilon =-\frac{N_{B}}{{N_{2}}^{2}}\frac{\left(Z_{0}+Z_{B}\right)}{Z_{d}A}$$

The error is inversely proportional to the loop gain, meaning that a higher loop gain results in a smaller error. The sensitivity of the detection winding (*Z*_d_) and the voltage gain of the auxiliary amplifier circuit play a significant role in increasing these parameters, while reducing the secondary winding’s internal resistance, leakage reactance, and load, leading to a smaller gain error. However, the inductive component in *Z*_0_ and *Z*_B_ has not been accounted for, so the actual value should be slightly larger.

## 4. Design of Wide-Ratio Self-Balancing Current Comparators

### 4.1. The Design of Winding Package Structure

The detection winding is uniformly wound in a non-directional manner around a primary core with extremely high initial permeability, terminated with shielded cables where the winding’s starting end connects to the core conductor while the terminating end connects to the cable shield and grounds. This configuration prevents current leakages from stray electric fields from entering the detection winding, thereby eliminating capacitive errors.

A first-layer magnetic shield core composed of four segments encloses the detection winding. When these four core pieces are stacked, an insulating layer must be inserted at one overlapping junction to prevent the assembly from forming a single-turn short circuit.

A copper shield layer is installed outside the first magnetic shielding layer. The primary compensation winding is uniformly wound over this copper shield, followed by a second-layer magnetic shield surrounding it. Both shielding layers share identical structures, but the second layer employs cold-rolled silicon steel laminations for its core. This material selection accounts for potential high local magnetic fields under heavy rated currents, as silicon steel offers higher saturation flux density for superior shielding performance.

**Figure 5:**
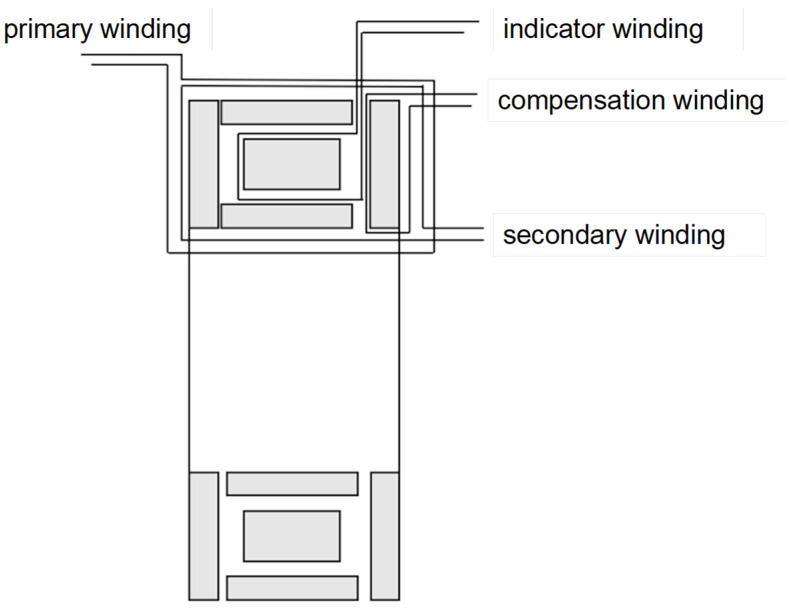
Structural diagram of the winding assembly for a wide-ratio self-balancing current comparator.

The compensation winding on the second magnetic shield connects to auxiliary circuits for core excitation, thereby inducing secondary currents. All core structures sequentially carry uniformly wound secondary and primary windings. Specific configuration is shown in the Figure 5.

### 4.2. Winding Structure Design

The wide-ratio self-balancing current comparator covers transformation ratios ranging from (5~5000) A/(5, 1) A, totaling 84 ratios. The (5~5000) A/1 A current ratio is achieved by cascading a 5 A/1 A self-balancing current comparator. The specific winding configurations are detailed in the Table 2.

In the winding process, it is essential to ensure that all ratio windings are uniformly distributed along the core as much as possible, as this method helps reduce magnetic errors.

### 4.3. Circuit Structure Design

The developed self-balancing standard current transformer connects the output of the detection winding to an electronic circuit, which then drives the compensation winding. The input of the electronic circuit is derived from the voltage signal of the detection winding, typically in the millivolt or microvolt range. The system achieves zero-flux when the voltage signal is minimized. In addition to requiring high gain, the circuit design must prioritize noise suppression and stability. Therefore, a multi-stage amplification approach is necessary, making the simultaneous achievement of high gain and high stability a potential challenge.

The high-gain amplifier circuit adopts a multi-stage amplification principle, where each stage consists of cascaded basic amplifiers. For each amplifier stage, the preceding stage acts as the signal source, while the subsequent stage serves as the load. Under loaded conditions, the total voltage gain of the multi-stage amplifier is the product of the gains of each individual stage. The input resistance is the equivalent resistance viewed from the input stage, and the output resistance refers to the equivalent resistance observed from the output stage.

**Figure 6:**
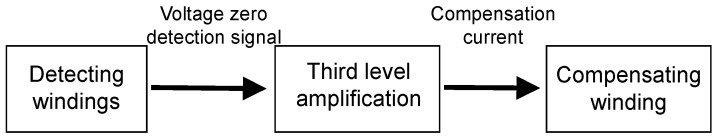
Schematic diagram of the overall structure of a high-gain, low-noise amplifier circuit.

For multi-stage amplifier circuits, it is essential to maximize the input resistance while minimizing the output resistance to prevent signal distortion and achieve a high voltage gain. This design employs a three-stage architecture, with the overall schematic illustrated is shown in the Figure 6.

The input stage achieves impedance matching between the amplifier and the signal source. The intermediate stage provides voltage gain, signal processing, frequency compensation, and other functions. The output stage features strong load-driving capability and delivers sufficient voltage and current output amplitude. Depending on different requirements, various coupling methods can be selected:

(a) Direct Coupling: When the input signal is zero, current and potential variations caused by temperature changes in the preceding stage are amplified progressively. This method can slowly amplify varying signals and facilitate integration, but results in interdependent static operating points and exhibits zero drift.

(b) RC Coupling: This blocks DC components, ensuring independent static operating points between stages. However, it cannot amplify slowly varying signals, has poor low-frequency characteristics, and is unsuitable for integration.

The design of high-gain feedback circuits should integrate the characteristics of different coupling methods with the requirements of multi-stage amplifier circuits. At the same time, to ensure that the circuit part is not affected by external magnetic and electric fields, we place the circuit board in a completely enclosed shielding box made of Permalloy material. The shielding box maintains effective grounding, isolating the electronic circuit part from the wire package part, which can effectively prevent external electromagnetic fields from interfering with the circuit signal.

The high-precision and wide-variable-ratio self-balancing current comparator has two advantages in design compared to traditional current comparators. Firstly, it simplifies the winding structure by replacing the large wire diameter and multi tap compensation winding structure in traditional current comparators and two-stage transformers with a single excitation winding. This reduces the size of the wire package and makes testing more lightweight. Secondly, the sensitivity of accuracy to the magnetic characteristics of the iron core is reduced, and cheaper silicon steel sheets can be used as auxiliary iron cores to reduce equipment production costs.

## 5. The Error Verification

**Figure 7:**
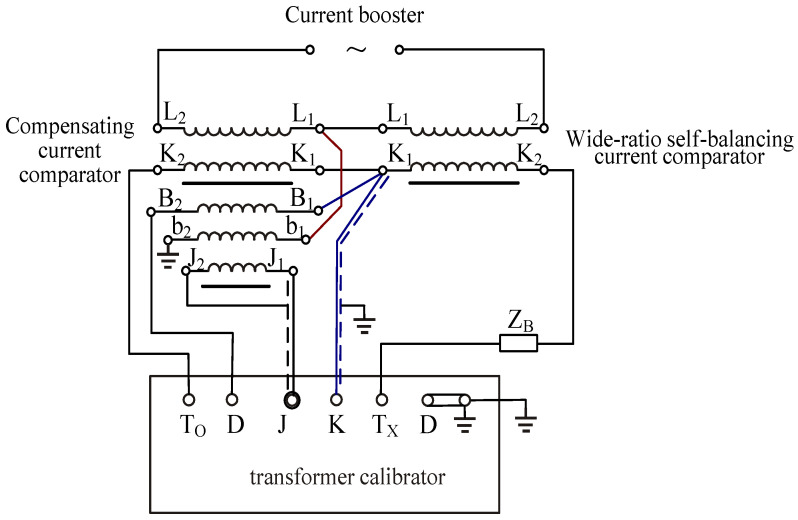
The circuit of current comparator verification for wide-ratio self-balancing current comparators. In Figure 7, L_1_L_2_ represents the primary ratio winding of the compensated current comparator; K_1_K_2_ denotes the secondary ratio winding of the compensated current comparator; B_1_B_2_ indicates the secondary compensation winding of the compensated current comparator; b_1_b_2_ refers to the primary compensation winding; J_1_J_2_ stands for the detection winding of the current comparator; T_O_ and T_X_ represent the working current circuit of the error measurement device; K and D correspond to the differential current circuit of the error measurement device; J signifies the zero-flux detection device of the error measurement device; Z_B_ represents the external secondary load of the current transformer under test.

Due to the fact that the wide-ratio self-balancing current comparator shares the same appearance as a single-stage current transformer but offers higher accuracy, a compensation-type circuit comparator is employed to verify the single-stage current transformer [29,30,31,32]. The experimental wiring principle is shown in Figure 7.

**Figure 8:**
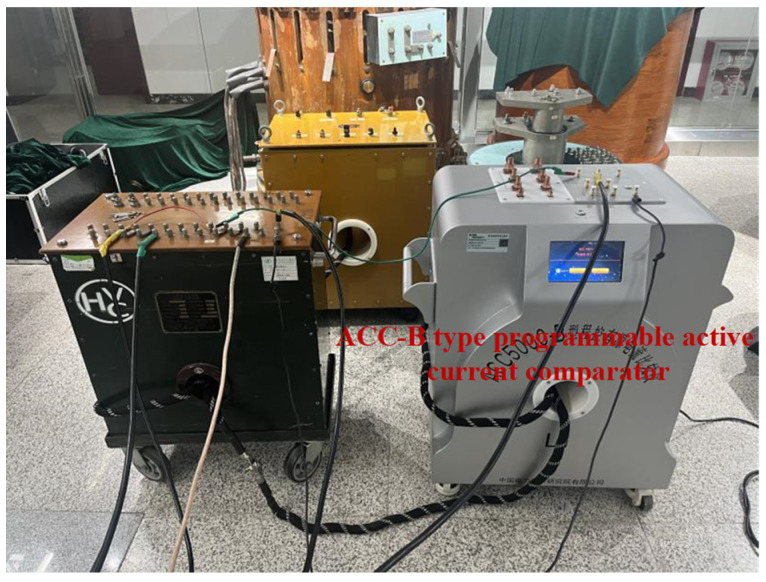
The calibration of a wide-ratio self-balancing current comparator using a current comparator.

The calibration test is shown in Figure 8.

The error verification was performed on a wide-ratio self-balancing current comparator under a frequency of 50 Hz, with a load of 0.2 Ω and 0.1 Ω at 20% of the rated current. Considering the scenario where the secondary rated ampere-turns differ, the measurement results are presented in the Table 3 [33].

The secondary current ratio of 1 A in the comparator is achieved through the cascading of a 5 A/1 A self-balancing comparator. Therefore, the comparator also requires individual calibration. The error measurement result is presented in the Table 4.

Taking a transformer with a ratio of 20 A/1 A as an example, when calibrated using a standard current transformer of 100 A/5 A, the obtained ratio error is −0.207 and the phase error is −2.456. The calibration result should be the sum of the errors for 20 A/5 A, which is −0.017 × 10^−6^ and −2.038 µrad, and for 5 A/1 A, the error is −0.157 × 10^−6^ and −1.196 µrad. The actual measurement results are in agreement with it.

According to the error results, it can be concluded that compared with the dual-stage current transformer with 0.001 level (5~5000) A/5 A, the accuracy level has been improved by two levels. Compared with the current comparator with an average of only 15 ratios, the number of ratios has increased by more than five times, and the load effect is less than 0.02 ppm and 0.1 μ rad. Since there is no need for manual zeroing for calibration tests, the test efficiency has been improved by 10 times.

## 6. Conclusions

This paper introduces a wide-ratio, high-precision, self-balancing current comparator, which detects the magnetomotive force (MMF) balance in the ratio windings through an electronic compensation circuit and automatically compensates for the excitation error of the current comparator. This ensures that the ampere-turn difference in the ratio windings remains at a minimal level, achieving a zero-flux state. By transforming conventional current transformers from traditional mutual inductance mode into the zero-flux mode of comparators, the accuracy class of current transformers is significantly improved.

Furthermore, by cascading a small 5 A/1 A self-balancing current comparator, high-precision transformation of 84 current ratios spanning (5–5000) A/(5, 1) A is achieved. Effective magnetic shielding and winding design keep the error of the wide-ratio self-balancing current comparator below 5 ppm, ensuring that all current ratios meet the 0.0005 accuracy class.

Additionally, this type of current comparator eliminates the need for manual ampere-turn balancing adjustments. In practical use, it operates as simply as a conventional current transformer, with straightforward wiring. When paired with a digital calibrator using the direct measurement method, it enables high-precision wide-ratio self-balancing. Since the compensation current is provided by the electronic circuit, the accuracy class of the measurement device is insensitive to secondary circuit impedance, offering strong load capacity. This makes it suitable for broader applications in measurement dissemination and current ratio transformation scenarios.

## Figures and Tables

**Table 1:** Shielding effectiveness under different frequencies and magnetic shielding structures.

Number of Shielding Layers	Induced Voltage (mV)		
	52.5 Hz	100 Hz	200 Hz
0	2.32	13.82	207.21
1	0.103	1.334	20.02
2	0.04	0.123	9.022
3	0.0016	0.017	0.102

**Table 2:** Wide-ratio self-balancing current comparator proportional winding turns configuration.

Secondary Turns	Primary Turns							
	L6-L	L5-L	L4-L	L3-L	L2-L	L1-L	2 Turns	1 Turns
160	5	8	20				400	
192	6							
200		10	25			250	500	
240	7.5	12	30	60		300	600	
252						315	630	
300		15		75	150		750	1500
320			40	80	160		800	1600
360							900	1800
400			50	100	200		1000	2000
480				120			1200	
500				125			1250	2500

**Table 3:** The winding turns configuration of a wide-ratio self-balancing current comparator.

Ratio	Secondary Winding Turns	Errors in PPM (0.2 Ω)		Errors in PPM (0.1 Ω)	
		ƒ (10^−6^)	δ (µrad)	ƒ (10^−6^)	δ (µrad)
5 to 5	160	−0.0103	−1.787	−0.0101	−1.562
6 to 5	192	0.003	−1.380	0.002	−1.273
10 to 5	200	0.132	−1.301	0.118	−1.116
30 to 5	240	0.030	−0.981	0.025	−0.732
40 to 5	320	0.062	−0.553	0.060	−0.541
50 to 5	400	0.165	−0.542	0.123	−0.418
75 to 5	300	0.002	−0.623	0.001	−0.601
120 to 5	480	0.087	−0.415	0.075	−0.402
125 to 5	500	0.065	−0.224	0.044	−0.198
315 to 5	252	0.047	−0.873	0.032	−0.791
900 to 5	360	0.022	−0.518	0.017	−0.499
3000 to 5	600	0.071	−0.421	0.065	−0.409
4000 to 5	800	0.085	−0.754	0.064	−0.722
5000 to 5	1000	0.065	−0.612	0.054	−0.601

**Table 4:** Error measurement results of a 5 A/1 A wide-ratio self-balancing current comparator.

Ratio	Secondary Winding Turns	Errors in PPM		Burden Resistance (Ohms)
		ƒ (10^−6^)	δ (µrad)	
5 to 1	120	−0.157	−1.196	0.2

## Data Availability

The original contributions presented in the study are included in the article, further inquiries can be directed to the corresponding author.
